# Societal preferences for funding orphan drugs in China: An application of the discrete choice experiment method

**DOI:** 10.3389/fpubh.2022.1005453

**Published:** 2022-12-12

**Authors:** Shuoyuan Tan, Yu Wang, Yuqing Tang, Rong Jiang, Mingsheng Chen, Haihong Chen, Fan Yang

**Affiliations:** ^1^School of Health Policy & Management, Nanjing Medical University, Nanjing, China; ^2^Center for Global Health, Nanjing Medical University, Nanjing, China; ^3^School of Medicine and Health Management, Tongji Medical College, Huazhong University of Science and Technology, Wuhan, Hubei, China; ^4^School of International Pharmaceutical Business, China Pharmaceutical University, Nanjing, China; ^5^The Research Center of National Drug Policy and Ecosystem, China Pharmaceutical University, Nanjing, China

**Keywords:** orphan drugs, discrete choice experiment, societal preference, willingness to pay, China

## Abstract

**Objectives:**

To explore whether a societal preference for orphan drugs exists in Chinese general public and to quantitatively measure the personal trade-off between essential attributes of orphan drugs through a discrete choice experiment.

**Methods:**

A labeled discrete choice experiment was employed to measure public preference. Six attributes (impact of diseases on life-years, impact of diseases on quality of life, availability of alternative drug treatments, annual cost per patient paid by medical insurance, expected increases in life-expectancy, and improvements to the quality of life) were identified through a literature review, experts' suggestions, and stakeholders' semi-structured interviews, then refined through a pre-survey. The current study used a D-efficient design to yield 27 choice sets divided into three blocks with nine questions containing the labeled treatment (either orphan drugs or common drugs). Information on sociodemographic characteristics and individual preferences was collected through a web-based questionnaire using convenience sampling. A mixed logit model was used to test societal preferences for orphan drugs over common drugs, while a binary logit model was used to measure the relative importance of each attribute in orphan drug access for the National Reimbursement Drug List and its willingness to pay.

**Results:**

A total of 323 persons participated in this study. Respondents largely had indifferent attitudes toward orphan drugs and common drugs. The binary logit model results showed that 5 of the 6 attributes were significant, except for the availability of alternative drug treatments. The most impacted factor was the annual cost per patient paid by medical insurance (β = −1.734, *odds ratio* [*OR*] = 0.177). Among non-economic attributes, the impact of diseases on life-years—with no treatment, the patient will die in the prime of life (β = 0.523, *OR* = 1.688, willingness to pay = 301,895)—was most concerning, followed by significant improvements to the quality of life (β = 0.516, *OR* = 1.676, willingness to pay = 297,773).

**Conclusion:**

The general public in China does not value rarity as a sufficient reason to justify special consideration in funding orphan drugs. When making orphan drug coverage decisions, the public prioritized the annual cost, disease severity, and drug effects.

## Introduction

Different from common diseases, rare diseases often have a low disease prevalence, difficulties in patient diagnosis and recruitment, and high treatment costs (1). However, because of the large size of the Chinese population, the low incidence of all rare diseases has resulted in a large number of patients. In China, the number of patients with rare diseases is >20 million and growing at a rate of 200,000 per year (2), placing an increasingly great disease burden on the society (3).

Medical insurance coverage is one of the most efficient ways to improve the accessibility of orphan drugs (4). As the resource is limited, however, policy-makers often face a dilemma between maximizing the total health benefits of society and achieving health equity while considering the minority group when making an orphan drug reimbursement decision (5). To resolve the conflict therein, priorities should be constructed to rank all alternative health technologies. Health technology assessment is the general approach to assess the value of drugs and medical devices. Traditionally, a cost-effectiveness analysis is used to test whether the extra cost paid for 1 quality-adjusted life-year (QALY) gained is under the willingness to pay (WTP) threshold. As for orphan drugs, their prices are usually high and the clinical evidence is weak despite the difficulty of conducting clinical trials. Through a cost-effectiveness approach, orphan drugs commonly have no chance of being prioritized over common disease drugs.

Rarity itself is not the criterion that leads to a prioritized reimbursement decision for orphan drugs (6). The value of orphan drugs is impacted by many value dimensions aside from cost and health outcome, such as unmet health needs, the severity of the disease, and the availability of alternative treatments (7). Although no consensus has yet been reached regarding the way to assess orphan drugs' value, multi-criteria appraisal methods have been applied in orphan drug reimbursement decision-making instead of cost-effectiveness approaches. The multi-criteria decision analysis (MCDA) method encompasses a group of assessment tools implied to assess the value of health technologies impacted by multi-dimensional factors. A number of attempts have been made to build or test these tools, such as reflective MCDA in the Catalan Health Service (8), the Evidence and Value: Impact of Decision-making (9), and other MCDA frameworks (10, 11), in different regions and from different perspectives to appraise orphan drugs. Among all the MCDA types, the discrete choice experiment (DCE) is one of the most commonly used methods to elicit individual preferences in the step of criteria weight measurement and becoming increasingly popular (12). Choice experiments could be suitable to reveal general preferences in health care priority settings (13) and can be completed by respondents themselves online (14).

The drug price negotiation is the way for brand name drugs (including most orphan drugs) to be covered by the Basic Medical Insurance in China. In the official document disclosed by the National Healthcare Security Administration in June 2022, price negotiation for orphan drugs was encouraged. However, no value assessment framework specific to orphan drugs has yet been established in China, which has provoked a wide discussion about the rationality of the medical insurance access mechanism. The question of what criteria are appropriate and acceptable for the general public should be considered when making a national health care reimbursement decision (15). This is especially the case for health technology, such as orphan drugs, because a single rare disease patient could consume the same amount of a medical resource needed by multiple common disease patients. As payers and beneficiaries of the medical insurance fund, the public is an orphan drug–reimbursement stakeholder. The societal preferences for orphan drugs have been measured in several western countries (16–18). Without other differences, no societal preference for rarity has been found across studies. The preference varied with the survey scenario setting, which reflected reimbursement policy and funding situation in each country, changed (16). No consensus has been reached on the list of orphan drug attributes and levels to be included in the trade-off study (18–20). As the heterogeneity of policy and subjectivity of preference, these studies only shed light on the value of research population and would not reflect preferences in other countries.

The objective of this study was to explore whether a societal preference for orphan drugs exists in China and to quantitatively measure the personal trade-off between essential attributes of orphan drugs in the Chinese general public.

## Methods

DCE is a quantitative research method designed to reveal stated preferences, which forces respondents to consider a trade-off between ≥2 alternatives in hypothetical scenarios. It allows the assessment of relative importance and the WTP for selected attributes by including a cost attribute (21). DCEs have been widely applied and have offered a great deal of priority-setting information in the health care sector (22, 23). By offering a detailed description of competing scenarios, DCE technique makes it easier to obtain the true preferences and leads to more precise weights than other weighting methods used in MCDA (24). Compared to other stated preference revealed methods, such as best-worst scaling (BWS), the stability, continuity and acceptability of DCE are better (25, 26).

DCE can be presented as labeled (such as orphan drugs vs. common drugs) or generic (such as treatment 1 vs. treatment 2). In the present study, a labeled DCE was employed because there is no official definition of rare diseases in China, so an attribute of orphan drugs (i.e., the rarity of the diseases) could not be accurately presented to the public. Unlike the generic experiment, the labeled experiment brands each alternative, containing information that is difficult to measure and will influence respondent's choices.

### Attributes and their levels

The use of qualitative research in DCE is an important step when selecting and determining the attributes and their corresponding levels (27). A 3-step approach was used to select attributes for the present study. First, an initial set of potential attributes and levels of orphan drugs was derived from a literature review. Some high-quality research reports of rare diseases and orphan drugs in China were also considered, such as Report of rare diseases in China (2018) (28) and the China rare disease drug accessibility report (2019) (29). Second, three experts on orphan drugs were invited to review the potential attributes and levels list. The experts were asked to check the comprehensiveness and rationality of selected attributes and levels as well as the accuracy of the description. Third, semi-structured interviews were conducted with 13 stakeholders, including rare disease patients, clinicians, specialists of orphan drug policy, policy-makers, and people working in the orphan drug industry. A refined list (shown in Supplementary material) was offered to the stakeholders, asking them to rate all attributes according to the importance in medical insurance access decision-making using a 10-point scale. Potential attributes and levels were not limited by the preliminary list. Each expert was encouraged to recommend any additional attributes he or she found relevant. The final rank of potential attributes was created after this round of reviews; the six most important attributes were finally incorporated, and the wording of all potential attributes and levels was further revised according to interview results and research team discussion (Table 1).

**Table 1:** Discrete choice experiment attributes and levels.

**Attribute classification**	**Attribute**	**Levels**	**Rationale for level**
Disease severity	Impact of diseases on life-years	- The disease does not decrease life-expectancy	Based on the European Conference on Rare Diseases and Orphan Products (ECRD), the Veneto Region's rare diseases registry, and a research report on the definition of the rare diseases in China (2021), the prime of life and childhood were chosen to represent the age of death
		- With no treatment, the patient will die in the prime of life (36–50 years)	
		- With no treatment, the patient will die in childhood (0–18 years)	
Disease severity	Impact of diseases on quality of life	- The patient does not face difficulties in everyday life, but strenuous activities may be contraindicated (e.g., sports)	Based on the Report of Rare Diseases in China (2018)
		- The patient may face difficulties in everyday life, but remains independent	
		- The patient needs assistance constantly	
Unmet needs	Availability of alternative drug treatments	- No other treatment exists	Based on the literature review and experts' suggestions
		- Other treatments are available, but their performances are limited	
		- Other treatments are available and their performances are good	
Financial burden	Annual cost per patient paid by medical insurance	−500,000 RMB	Referred to the reimbursement cap line of the Basic Medical Insurance in China, maximum and minimum values were extracted from 2 representative cities
		−300,000 RMB	
		−150, 000 RMB	
Drug treatment effectiveness	Expected increases in life-expectancy	- Drug treatment increases life-expectancy by 10 years	Based on the evidence of the effectiveness of orphan drugs and experts' opinions
		- Drug treatment increases life-expectancy by 2 years	
		- Drug treatment has no impact on life-expectancy	
Drug treatment effectiveness	Improvements to the quality of life	- Significant improvement	Based on the usual activity domains of EQ-5D (e.g. work, study, housework, family, and leisure activities)
		- Slight improvement	
		- No improvement	

### Experimental design

The experiment design employed Ngene version 1.1 (ChoiceMetrics, Sydney, Australia) to define the combination of levels for the choice set and avoid the logically impossible ones. The experiment was conducted among the general population following the good practice guidelines of DCE (30, 31) in two steps: a pre-survey and a formal survey. To improve the clarity of the survey and questionnaire feasibility for respondents, 135 eligible respondents were recruited for the pre-survey to test the questionnaire and collect a priori information about the value of the attributes. The pre-survey was generated using an orthogonal main effects design. Feedback from the pre-survey led to further wording adjustment of the questionnaire; in this way, the questionnaire was updated to its final version. A D-efficient design was incorporated in the formal survey, and the results of the pre-survey were adopted as prior values. A D-efficient design was used to minimize standard errors and parameter prior variances in DCE (32).

**Figure 1:**
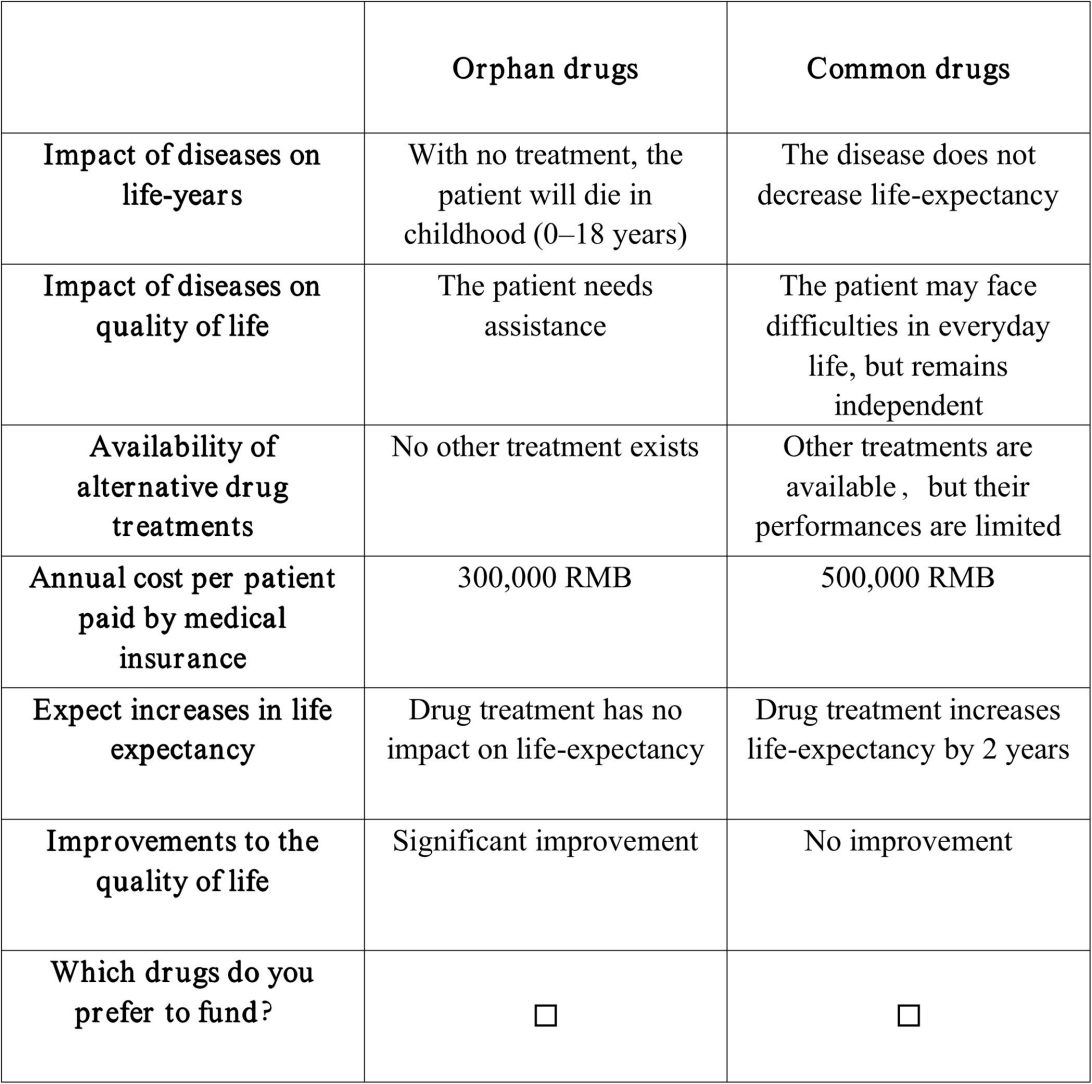
Example of choice set.

The final number of choice situations was limited to 27 situations, which were divided into three blocks with nine questions in each and consisted of the labeled orphan drugs and common drugs. The blocking method was used to limit the burden of respondents, which also satisfied efficient design criteria (31). The choice set did not represent any specific existing drugs. The survey did not offer the opt-out option because the options in our DCE were a fair reflection of medical insurance drug access in China. Furthermore, a final choice set (the 10th choice set), which was the same as the second choice set, was added. The repeated choice set was used as an internal consistency check of the questionnaire to ensure that respondents were engaged in the experiment and taking it seriously. The 10th choice set was subsequently dropped from the final statistical analysis. Figure 1 shows an example of the choice set as presented to the respondents.

### Study sample and survey administration

According to the International Society for Pharmacoeconomics and Outcomes Research Task Force Report on DCE construction, the sample size of DCE is decided by the object of the study and the number of attributes and levels (33). When blocking a DCE questionnaire into different versions, >20 participants per version are rarely required to estimate reliable models (34). It was reported that the mean sample size for conjoint analysis studies in health care published between 2005 and 2008 was 259, with nearly 40% of DCEs having sample sizes of 100–300 respondents (35). The following formula used to calculate the minimum sample size in DCE was also considered: n = (500^*^c)/(t^*^a), where “c” represents the largest number of levels for an attribute, “t” represents the number of choice sets in a block, and “a” represents the number of alternatives (36). While there was limited consensus on appropriate sample sizes for DCE studies, a sample size of 300 participants in total and 100 participants per version would be sufficient for a reliable statistical analysis.

A convenience sampling method was used in this study and those who met all the following criteria were included: (1) Chinese citizens aged ≥18 years, (2) individuals with no diagnosis of a rare disease, (3) individuals who participated in the Basic Medical Insurance in China, and (4) individuals without cognitive impairment who were willing to sign the informed consent form and finish the questionnaire on their own.

If the participants had no prior knowledge or cognitive basis, their preferences were assumed to be unstable and constructed rather than just revealed in the process of answering choice-related questions (37). As the funding issue of orphan drugs is beyond the common sense of the public, the questionnaire started with a brief introduction of rare diseases and orphan drugs as well as the disease burden of both rare diseases and common diseases. Socioeconomic characteristics of the respondents (e.g., sex, age, education) were collected. A profile that clearly defined all attributes and levels and an example of the choice set were shown to respondents. Each respondent was asked to make reimbursement trade-offs between the paired alternatives. Before making decisions, the respondents were informed that the Basic Medical Insurance funding could not cover all drugs because of limited health care budgets. They were asked to place themselves in a hypothetical situation as decision-makers and to prioritize the drugs that should be reimbursed. It was specified that choosing an alternative meant that resources could not be allocated to the other one. The 10 choice sets were presented to the respondents, following the description of the hypothetical situation.

### Statistical methods

Descriptive statistics were used to present the demographic characteristics of the respondents. The random utility model provided the theoretical underpinning for analysis of the DCE data (5). In a choice set, it assumed that individuals chose a certain alternative that yielded a higher utility to them over the other one. Utility was calculated using the following formula:


$$\begin{array}{l} U_{njt}=V_{njt}+\varepsilon_{njt}=X_{njt}\beta +\varepsilon_{njt} \end{array}$$

Where *U*_*njt*_ represents the utility respondent n obtained from alternative j on choice set t, which was composed of a systematic unit (*V*_*njt*_) and a random unit (ε_*njt*_); *X*_*njt*_ represents the explanatory vector of the attribute; and β represents the coefficient vector of the corresponding preference value, the size and significance of which is the weight of each level of each attribute. In other words, the coefficients of the model can be interpreted to define the relative importance that the respondents gave to the movement of any given attribute from the reference level to a different level (38). Apart from the attributes and levels, there was an alternative specific constant, which reflected whether the public had a preference for the label of orphan drugs or common drugs.

The DCE data were analyzed using a mixed logit model and binary logit model. The mixed logit model was used to test whether the Chinese public preferred orphan drugs over common drugs and whether the listed attributes were important factors for drug funding decisions, while the binary logit model was used to measure the relative importance of each attribute in orphan drug access for National Reimbursement Drug List (NRDL) and its WTP. The response variable was a binary (0/1) dependent discrete variable where “1” represented the alternative chosen and “0” represented the one not chosen. Except for the continuous variables of attributes, including the annual cost per patient paid by medical insurance and expected increases in life-expectancy, other attributes were considered as categorical variables. An example of the categorical variable code is listed as follows: a = reference case [0], b = 1, and c = 2. Furthermore, the WTP was also calculated, which represented the marginal utility respondents were willing to pay for a particular change in attribute level. Finally, an exclusion criterion was applied to remove the questionnaires completed in ≤60 s. All statistical analyses were performed using STATA version 16.0 (StataCorp LLC, College Station, TX, USA).

## Results

### Respondent characteristics

A total of 412 individuals participated in the formal survey, 346 questionnaires were initially considered valid (valid response rate, 83.98%), but 323 questionnaires were finally included in the analysis after excluding those completed in ≤60 s. The sociodemographic characteristics of the study sample are presented in Table 2. Women represented 69.66% of the respondents, and 37.77% of the respondents were aged 35–44 years. A Bachelor's degree or higher was held by 72.13% of the respondents, and 78.64% of the respondents self-reported that they were healthy. A relatively large portion of participants were married (74.61%) and most of participants had children (69.97%). The largest group (34.37%) had a family income of 100,001–200,000 RMB per year.

**Table 2:** Sociodemographic characteristics of the study sample.

**Characteristic**	***N* (%)**
**Gender**	
Female	225 (69.66%)
Male	98 (30.34%)
**Age**	
18–24	50 (15.48%)
25–34	72 (22.29%)
35–44	122 (37.77%)
45–50	35 (10.84%)
51–60	32 (9.91%)
60+	12 (3.72%)
**Education status**	
Elementary school and below	2 (0.62%)
Middle school	10 (3.10%)
High school	27 (8.36%)
College	51 (15.79%)
University	138 (42.72%)
Postgraduate	95 (29.41%)
**Self-reported health status**	
Very poor	1 (0.31%)
Relatively poor	7 (2.17%)
Average	61 (18.89%)
Relatively good	151 (46.75%)
Very good	103 (31.89%)
**Marital Status**	
Unmarried	74 (22.91%)
Married	241 (74.61%)
Other	8 (2.48%)
**Household composition**	
With children	226 (69.97%)
Without children	97 (30.03%)
**Family Income(**¥**per annum)**	
≤ 500,00	29 (8.98%)
500,01–100,000	67 (20.74%)
100,001–200,000	111 (34.37%)
200,001–500,000	93 (28.79%)
>500,000	23 (7.12%)

### DCE results

The results of the mixed logit model are presented in Table 3. A positive coefficient means that the corresponding level has a higher utility than the reference level, and the probability of being chosen would be higher in this context. The estimated means for orphan drugs (β = −0.536, *p* = 0.351) was not statistically significant, meaning that respondents had no preference to fund orphan drugs over common drugs considering only the rarity of the disease when controlling for the defined attributes differences. The estimated coefficients were all statistically significant (*p* < 0.05), indicating that the 6 selected attributes in the experiment were all important factors influencing the respondents' reimbursement choice between orphan drugs and common drugs. As excepted, the public preferred to reimburse the drugs that treated severe diseases rather than moderate diseases, that had no other alternative treatments available rather than had alternatives, that were cheaper rather than expensive, and that produced more clinical outcomes rather than were less effective.

**Table 3:** Results of Mixed logit model and Binary logit model.

**Attribute**	**Mixed logit**			**Binary logit**		
	**β**	***SE***	**95% CI**	**β**	***OR***	***OR-*95% CI**
**Impact of diseases on life-years**						
The disease does not decrease life-expectancy	–	–	–	–	–	–
With no treatment, the patient will die in the prime of life (36–50 years)	0.40*	0.09	0.22~0.59	0.52*	1.69*	1.29~2.21
With no treatment, the patient will die in childhood (0–18 years)	0.49*	0.09	0.30~0.67	0.50*	1.65*	1.27~2.14
**Impact of diseases on quality of life**						
The patient does not face difficulties in everyday life, but strenuous activities may be contraindicated (e.g., sports)	–	–	–	–	–	–
The patient may face difficulties in everyday life, but remains independent	0.15*	0.06	0.03~0.26	0.28*	1.33*	1.07~1.65
The patient needs assistance constantly	−0.12*	0.06	−0.24~-0.01	0.19	1.20	0.97~1.49
**Availability of alternative drug treatments**						
No other treatment exists	–	–	–	–	–	–
Other treatments are available, but their performances are limited	−0.16*	0.06	−0.28~−0.05	−0.03	0.97	0.79~1.19
Other treatments are available and their performances are good	−0.21*	0.06	−0.32~−0.10	−0.19	0.83	0.674~1.01
**Annual cost per patient paid by medical insurance**	−0.64*	0.24	−1.11~−0.16	−1.73*	0.18*	0.10~0.32
**Expected increases in life-expectancy**	0.07*	0.01	0.05~0.09	0.06*	1.06*	1.04~1.09
**Improvements to the quality of life**						
No improvement	–	–	–	–	–	–
Slight improvement	0.17*	0.08	0.01~0.33	0.46*	1.58*	1.28~1.95
Significant improvement	0.52*	0.07	0.39~0.66	0.52*	1.68*	1.38~2.04
**Constant**	−0.54*	0.58	−1.66~0.59	−0.19	–	−0.57~0.19
**AIC**	3619.814			3747.493		
**BIC**	3775.161			3813.216		

β, coefficient; SE, standard error; CI, confidence interval;

* P < 0.05.

The results of binary logit model are shown in Table 3. The results showed that the coefficients were significant (*p* < 0.05) for five out of the six attributes, including the impact of diseases on life-years, impact of diseases on quality of life, annual cost per patient paid by medical insurance, expected increases in life-expectancy, and improvements to the quality of life. Meanwhile, societal preference was not influenced by whether there were other available treatments or not.

The annual cost per patient paid by medical insurance significantly impacted participants' choices [β = −1.734, *odds ratio* (*OR*) = 0.177]. With a higher cost, the possibility of being chosen was lower. It can be clearly seen that the disease's impact on life-years was the most impacted non-economic factor. The participants were 1.688 [95% confidence interval (CI) = 1.290–2.207, β = 0.523] times more likely to fund orphan drugs that treated rare disease patients who would die in the prime of life than those that treated rare diseases with no impact on life-years. Additionally, the participants were sensitive to drug improvements to the quality of life. A significant improvement increased the odds of preferring to fund an orphan drug by 1.676 times (95% CI = 1.378–2.038, β = 0.516) compared to no improvement. All else being equal, the odds of successfully listing an orphan drug in NRDL increased by 1.062 (95% CI = 1.039–1.085, β = 0.060) for 1 additional year of survival and by 1.328 (95% CI = 1.071–1.646, β = 0.284) for treating a patient who had difficulties in daily life but remained independent. The results revealed that the respondents would not want to prioritize the Basic Medical Insurance fund on orphan drugs according to the unavailability of alternative treatments or the insurance annual cost of the drug. There was a preference toward drugs that treated severe rare diseases or that had a good effect on the improvement of life-years or quality of life.

### Results of WTP

**Figure 2:**
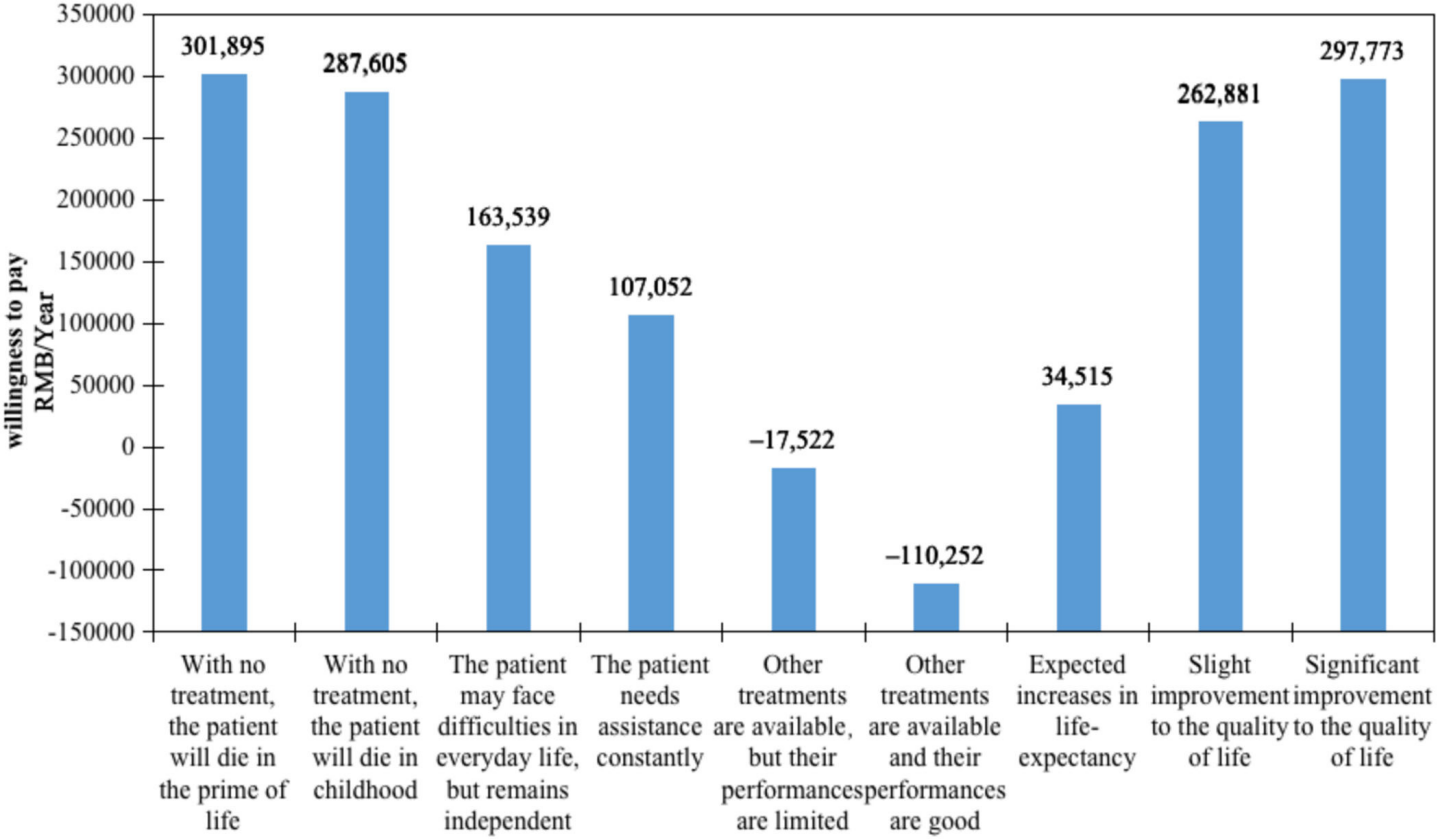
Willingness to pay for attributes of orphan drugs.

The WTP values shown in Figure 2 indicate the rate at which participants' trade-off of drug costs increased according to gains in other criteria. The comparison of each attribute is presented in economic value form, representing the relative importance of each attribute level. The WTP analysis demonstrated that the impact of diseases on life-years was the most valued attribute. Participants were willing to spend 301,895 RMB per year for rare disease patients who would die in the prime of life with no treatments and 287,605 RMB per year for rare disease patients who would die in childhood rather than patients whose life-years would not decrease. Drug improvements to the quality of life were revealed to be another essential attribute. All else being equal, participants were willing to pay 297,773 RMB to improve patients' quality of life significantly. Moreover, the WTP for 1 year of life expectancy gained was 34,515 RMB.

## Discussion

To our knowledge, this paper is the first study to quantitatively investigate individual preferences of the Chinese public and their trade-offs to have the Basic Medical Insurance fund orphan drugs. As the study was conducted on a convenience sample of the general public in China, we consider it to be a pilot MCDA study. The preferences revealed by this study could be used as a priori information for future Bayesian designing of DCEs. The present research showed that public had an indifferent attitude toward orphan drugs and common drugs. This finding aligned with other preferences revealed in studies conducted in other countries (16), which found no evidence of a preference to fund high-cost treatments for rare diseases on the basis of rarity alone. This was also in line with National Institute for Health and Clinical Excellence (NICE)'s evaluation philosophy, which highlights that the “rule of rescue” would not be applicable in an economic evaluation (39), and the criteria for being a highly specialized technology include not only the rarity of the disease but also the severity of the disease and the effectiveness of the drug as well (40). There has always been debated about implementing a higher WTP threshold for orphan drugs (41). In China, no WTP threshold is set specifically for orphan drugs right now and, according to the results of the present study, no evidence exists supporting its necessity. To get an orphan drug listed for reimbursement, multiple criteria should be considered rather than just rarity alone.

Despite all this, slightly more (57%) respondents were observed to prefer funding orphan drugs over common drugs. The first possible explanation for this trend is that the Chinese public has a tendency for altruism—in other words, some subjects were willing to sacrifice part of their own payoff to help people in need (42). Meanwhile, altruism is also an inherent quality in Chinese culture. This point had been demonstrated by the feedback from some of the respondents. The second possible reason was that the results of a prosocial behavior study designed in a laboratory experiment might be more positive than those from a field experiment (43).

Furthermore, this study measured the relative importance of the orphan drug value elements to society. The DCE results suggested that all these attributes mattered significantly from the societal perspective, except for the availability of alternative drug treatments. The results of the experiment offered some hints on priority setting for orphan drugs to be listed in NRDL. Attention should be paid to the annual cost per patient paid by medical insurance, which outweighed all other influences. This finding was consistent with findings of other DCEs conducted from the perspective of decision-makers and patients in European countries (38, 44). There was great evidence that the general public would prioritize orphan drugs that treat severe diseases as well as confer great health gains (17). The top 2 WTP values revealed that the public were willing to pay for orphan drugs that treated diseases from which patients would die in the prime of their life or that could significantly improve patients' quality of life, highlighting the importance of disease severity and drug effect. These two criteria are often related to QALY and presented as quality of life and life-years. Another DCE study have indicated the significance of disease severity and drug effect from different population and in different countries (19). Some studies even argued that these two criteria should be prioritized over cost (20). The availability of alternative drug treatments is a debatable attribute. In the present study, the public showed no preference toward it, yet this was a key factor emphasized by experts in the semi-structured interviews in this study. A similar study from the perspective of the U.K. public also revealed the same result as ours (18), while other studies have demonstrated that the existence of alternative treatments (unmet needs) is an influential factor of societal preferences (19, 45). The difference might result from disparate perspectives of thinking and difficulty in understanding the attribute for the general public. A study also found this disparity existed between the general public and the experts, which was attributed to differences in knowledge and scope (46).

The present study has some limitations. First, due to the coronavirus disease 2019 pandemic, the experiment was conducted online instead of face-to-face. Although it has been proved effective to reveal individual preferences by an online method, the face-to-face method would still have been preferred so that the researchers could catch all feedback from respondents and provide aid when respondents found it difficult to understand. Second, the sample employed in the present study might not perfectly represent the general public in China. However, the results of this study as a pilot study are still considered to have a certain degree of generalizability.

All the evidence gathered herein indicated the necessity to consider multi-level criteria in the orphan drug value assessment process in China. Societal preferences should not be the only evidence used to resolve complex ethic issues regarding orphan drug priority setting. National health resource allocation decisions should be deliberative, taking both multi-stakeholders' preferences and ethical principles into consideration. To establish a specific value assessment framework for orphan drugs, future studies should be conducted in broader samples and multiple stakeholders, including but not limited to the general public, patients, physicians, decision-makers, policy researchers, and people working in the orphan drug industry. Meanwhile, future experiments should explore better DCE designs. Interviewer-dominated face-to-face experiments should be implemented to obtain less biased results. Different colors could be set to different levels, reducing the complexity in the DCE choice task (47). Lastly, more comprehensive criteria should be considered in future studies, such as the quality of clinical evidence and uncertainty of drug and opportunity costs (48). However, the key aspect is to control the increasing complexity of the trade-off task caused by the increasing number of attributes and levels.

## Conclusion

To conclude, the results of the present study shed light on the possibility of constructing a MCDA framework using the DCE method to assess the value of orphan drugs, deviating from the traditional cost-effectiveness analysis process. The general public in China does not value rarity as a sufficient reason to justify special consideration in funding orphan drugs. When making coverage decisions, the public prioritized the annual cost, disease severity, and drug effects.

## Data availability statement

The original contributions presented in the study are included in the article/Supplementary material, further inquiries can be directed to the corresponding authors.

## Ethics statement

The studies involving human participants were reviewed and approved by the Ethics Committee of Nanjing Medical University. The patients/participants provided their written informed consent to participate in this study.

## Author contributions

ST contributed to collection and assembly of data, data analysis, and manuscript writing. YW and YT contributed to data analysis and manuscript review. RJ and MC contributed to manuscript review. HC contributed to data collection, manuscript review, and administrative support. FY contributed to study design, manuscript writing, and administrative support. All authors approved the final version of the manuscript before its submission.
