# Supplementary material for: Societal preferences for funding orphan drugs in China: An application of the discrete choice experiment method
Source: Front Public Health. 2022 Dec 12;10:1005453. doi: 10.3389/fpubh.2022.1005453 (PMC9790908; doi:10.3389/fpubh.2022.1005453)
Supplement: Supplementary file 1 [file Table_1.DOCX]

**Supplementary material:** all potential attributes offered to the stakeholders

| **Attribute classification** | **Attribute** | **Levels** | **Rationale for level** |
| --- | --- | --- | --- |
| Characteristics of influenced patients | The morbidity of diseases | - < 1/100,000 | Based on the Research Report on the definition of Rare Diseases in China |
|  |  | - < 1/10,000 |  |
|  |  | - > 5/10,000 |  |
|  | Age at Onset | - < 18 years | Expert opinion |
|  |  | - ≥ 18 years |  |
| Disease severity | Impact of diseases on life-years | - The disease does not decrease life-expectancy | Based on the European Conference on Rare Diseases and Orphan Products (ECRD), the Veneto Region’s rare diseases registry, and a research report on the definition of the rare diseases in China (2021), the prime of life and childhood were chosen to represent the age of death |
|  |  | - With no treatment, the patient will die in the prime of life (36–50 years) |  |
|  |  | - With no treatment, the patient will die in childhood (0–18 years) |  |
| Disease severity | Impact of diseases on quality of life | - The patient does not face difficulties in everyday life | Based on the Report of Rare Diseases in China (2018) |
|  |  | - The patient may face difficulties in everyday life, but remains independent |  |
|  |  | - The patient needs assistance constantly |  |
| Unmet needs | Availability of alternative drug treatments | - No other treatment exists | Based on the literature review |
|  |  | - Other treatments are available，but their performances are limited |  |
|  |  | - Other treatments are available and their performances are good |  |
| Financial burden | Annual cost per patient paid by medical insurance | - 500,000 RMB | Referred to the reimbursement cap line of the Basic Medical Insurance in China, maximum and minimum values were extracted from 2 representative cities |
|  |  | - 300,000 RMB |  |
|  |  | - 150,000 RMB |  |
| Drug Safety | Adverse drug effects | - Treatment may cause adverse effects leading to death or disability | Based on the literature review |
|  |  | - Treatment may cause adverse effects with moderate impact on health which will disappear when patient stops taking |  |
|  |  | - Treatment may not cause adverse effects |  |
| Drug treatment effectiveness | Expected increases in life-expectancy | - Drug treatment increases life-expectancy by 10 years | Based on the evidence of the effectiveness of orphan drugs |
|  |  | - Drug treatment increases life-expectancy by 2 years |  |
|  |  | - Drug treatment has no impact on life-expectancy |  |
| Drug treatment effectiveness | Improvements to the quality of life | - Significant improvement | Based on the usual activity domains of EQ-5D (e.g. work, study, housework, family, and leisure activities) |
|  |  | - Slight improvement |  |
|  |  | - No improvement |  |
| Evidence quality | Certainty regarding available evidence | - Sufficient evidence | Based on the literature review |
|  |  | - Some evidence |  |
|  |  | - Controversial evidence |  |
